# Early detection of maternal deaths in Senegal through household-based death notification integrating verbal and social autopsy: a community-level case study

**DOI:** 10.1186/s12913-014-0664-4

**Published:** 2015-01-22

**Authors:** Mosa Moshabela, Massamba Sene, Ingrid Nanne, Yombo Tankoano, Jennifer Schaefer, Oumulkhairy Niang, Sonia Ehrlich Sachs

**Affiliations:** Department of Rural Health, School of Nursing and Public Health, University of KwaZulu-Natal, Science Drive, Howard College, Fourth Floor, George Campbell Building, Durban, 4001 South Africa; Millennium Promise, Columbia University, Dakar, Senegal; Millennium Development Goal Centre, Columbia University, Dakar, Senegal; Earth Institute, Columbia University, New York City, USA; School of Public Health, Tropical Medicine and Rehabilitation Sciences, James Cook University, Townsville, Australia

**Keywords:** Maternal mortality, Verbal autopsy, Social autopsy, Community health worker, Quality improvement, Quality of care, Pathway to survival, mHealth, Africa

## Abstract

**Background:**

Reliable detection of maternal deaths is an essential prerequisite for successful diagnosis of barriers to care and formulation of relevant targeted interventions. In a community-level case study, the use of household-level surveillance in Senegal unveiled an apparent increase in maternal deaths, which triggered a rapid-cycle collaborative response to implement a multipronged set of quick-win and sustained interventions intended to improve quality care.

**Methods:**

Part of a multi-country effort, the Millennium Villages Project is implementing a routine community-level information system in Senegal, able to detect maternal deaths in real-time and uncover clinical and social factors contributing to mortality. Within this geographically demarcated area of approximately 32 000 inhabitants, with a well-structured health system with patient referral services, deaths were registered and notified by community health workers, followed by timely verbal and social autopsies. Using the *Pathway to Survival* conceptual framework, case analysis and mortality reviews were conducted for evaluation and quality improvement purposes.

**Results:**

The estimated maternal mortality rates rose from 67/100000 births in 2009 (1 death), to 202/100000 births in 2010 (3 deaths) and 392/100000 births (5 deaths) in 2011. Although absolute numbers of maternal deaths remained too small for robust statistical analysis, following verbal autopsy analyses in 2011, it became evident that an unexpectedly high proportion of maternal deaths were occurring at the referral hospital, mostly post-Caesarian section. Inadequate case management of post-partum haemorrhage at the referral hospital was the most frequently identified probable cause of death. A joint task team systematically identified several layers of inefficiencies, with a potential negative impact on a larger catchment area than the study community.

**Conclusions:**

In this study, routine community-based surveillance identified inefficiencies at a tertiary level of care. Community-level surveillance systems that include pregnancy, birth and death tracking through household visits by community health workers , combined with verbal and social autopsy can identify barriers within the continuum of maternal care. Use of mHealth data collection tools sensitive enough to detect small changes in community-level mortality trends in real-time, can facilitate rapid-cycle quality improvement interventions, particularly when associated with social accountability structures of mortality reviews.

## Background

Pregnancy can be a risky undertaking, particularly the occurrence of maternal complications during childbirth. In 2010, an estimated 1700 maternal deaths occurred in Senegal, with a country-wide maternal mortality ratio (MMR) of 320 deaths per 100,000 live births [1]. The lifetime risk of death from pregnancy-related causes for a woman in Senegal was reported as 1 in 60 in 2010, with a sixth of all deaths of women aged 15–49 thought to be related to pregnancy [1]. Furthermore, Senegal is among many countries in sub-Saharan Africa declared unlikely to meet the Millennium Development Goal Five (MDG5) target of a three-quarter reduction in maternal mortality between 1990 and 2015 [2,3]. Tracking progress in MDG5 is hindered by the limited reliability of data regarding maternal deaths, including weaknesses in detection and classification of maternal deaths [1,4,5]. Reducing maternal mortality requires understanding of who, where, when and how maternal deaths occur [6], which relies on accurate vital statistics data. Unfortunately, vital events registration is often poor in areas of highest maternal mortality, including Senegal, where only an estimated 55% of births were recorded in 2008 [6].

Every case of maternal death offers a unique opportunity to understand and mitigate weaknesses in the continuum of care, encouraging interventions that are better tailored to local circumstances, and targeted at the most critical parts of the health system [7,8]. Notably, perinatal mortality reviews have been instrumental in highlighting the poor quality of care within the formal health system, a major factor contributing to high mortality rates in low and middle-income countries, including Senegal [8-10]. Procedurally, maternal death reviews are typically conducted using health facility-based records [11], and therefore largely detect barriers to care only for those patients who successfully reach the facility [9,10]. Community-based mortality reviews allow for a more comprehensive and participatory assessment of perinatal deaths than facility-based reviews [9]. Verbal autopsies (VA) have been used effectively at both facility and population level for the identification of maternal deaths, and the causes thereof. Verbal autopsies use a structured, standardised questionnaire to determine probable cause of death [12].

Historically, VA had to be independently reviewed by two physicians, which was a time-consuming and expensive process, and as result, VA use was restricted largely to research and demographic surveillance sites [13]. Currently, there are growing efforts to position VA as part of routine surveillance. The World Health Organisation (WHO) has recently simplified its VA standards to increase utility, cost-effectiveness and suitability for use with software that incorporates automated ascertainment of death algorithms (13). There has also been progressive adoption of social autopsy, which extends emphasis from the biomedical causes of death to include social, economic, cultural, and health system factors. These shifts have given way to community-based mortality reviews, which allow for a more comprehensive and participatory assessment of perinatal deaths [9]. As a result, integrated verbal and social autopsy (VASA) tools have proven particularly useful at a community level given their comprehensive scope, able to highlight social barriers in the pathways of care in addition to medical factors [14].

Conceptually, the Three Phases of Delay [15], a) Seeking care, b) Reaching facility and c) Receiving appropriate treatment, is the predominant approach used for maternal death reviews in obstetric care. The Three Phases of Delay are used to map out barriers occurring before and after arrival in the health facility, with a particular emphasis on delays often identified through maternal death reviews using facility-based records. An alternative, but similar framework is the Pathway to Survival [16], which incorporates the key steps of, a) Recognizing illness or health problem, b) Seeking assistance, c) Referring appropriately, and d) Receiving adequate treatment. These frameworks take a holistic view of health care, which includes households, community, referral systems and health care facilities. The Pathway to Survival Framework was developed for child survival [17], but remains equally relevant in the documentation of maternal survival along the continuum of care. Most importantly, the Pathway to Survival is a framework originally designed, and therefore better-suited, to support community participation and empowerment [14].

In this paper, we present a case study describing a series of five maternal deaths in north-western Senegal in 2011. The case study was undertaken as part of mortality tracking and response activities conducted across all MVP sites. These activities are integrated into routine quality improvement processes, and designed to strengthen health care delivery systems through a better understanding of who, where, when, how and why deaths occur [7], using the Pathway to Survival Framework. The nested case study was conducted as part of the Millennium Villages Project (MVP), an integrated rural development project designed to achieve the Millennium Development Goals (MDGs) in varied low-income rural African settings by the year 2015. The ten-year project operates at more than a dozen rural sites in countries throughout sub-Saharan Africa, including core sites in ten countries. The MVP strategy, described in detail elsewhere [18,19], involves simultaneous and integrated investments in the five key sectors of health, agriculture and environment, education, infrastructure, and business development.

Data collection on births and deaths at MVP sites is embedded within routine activities, such as Community Health Worker (CHW) household visits and routine care of pregnant women, with comprehensive VASA conducted for deaths of women of reproductive age. Reproductive age mortality studies (RAMOS) attempt to reduce misclassification by identifying and characterising causes of death for *all* women of reproductive age, not just those known to be pregnant [6,20]. Mobile Health (mHealth) applications were utilised to support CHW household level activities and VASA data collection. Increasingly, mHealth applications are being successfully utilized in rural African settings [21,22]. Such tools may improve timeliness and consistency of documentation at a community level, including mortality tracking [20,23]. In addition, we used a quality improvement rapid cycle change model to respond to findings of health care inefficiencies [24]. Using a rapid cycle change model in health care involves a response to a given medical problem through (1) team formation; (2) study of the problem; (3) development and roll-out of an intervention plan; and (4) evaluation of the intervention.

## Methods

### Study design

The Senegal MVP case study, involving a case-series analysis and subsequent intervention for maternal deaths, was prompted by an apparent increase in maternal mortality trends and the findings of integrated VASA. The case-series included all recorded maternal deaths from 2011, and can be considered opportunistic in that it was nested within routine data collection and feedback activities conducted by CHWs as part of the much broader ten-year MVP study. Routine vital statistics tracking and VASA began prior to the study period and continued beyond. Maternal mortality trends are presented as numbers of recorded maternal deaths from the start of 2007 to the end of 2012.

**Figure 1:**
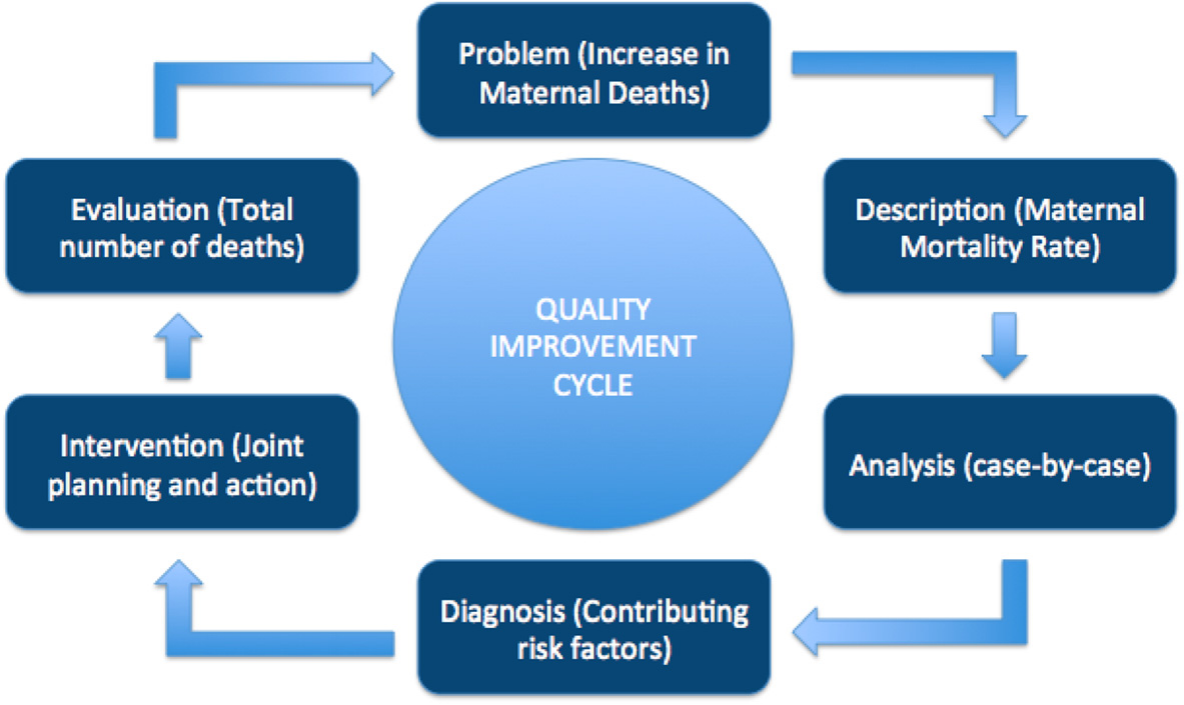
**Modified rapid cycle change model for quality improvement.**

Cases of maternal death were identified via active household-level surveillance of pregnancies, births and deaths, using mHealth platform Childcare+. A standardized VASA questionnaire was used to collect descriptive case data retrospectively following the death of any women aged 12–49 who lived within the geographical study area. Maternal deaths were defined as deaths of women while pregnant or within 42 days post-delivery, regardless of cause of death. Cases were assessed individually with VASA, and then collectively as part of a case-series analysis. The Pathway to Survival framework was used to identify areas of failure within the care continuum, and a rapid cycle change model (Figure 1) was used as a conceptual framework to guide quality improvement discussions and interventions.

### Study setting and participants

The study was conducted in a geographically demarcated area in northwestern Senegal, at one of the core MVP study sites. The densely-populated study area consists of a cluster of coastal villages and is home to approximately 32,000 individuals. The entire community received the complete integrated package of MVP interventions. When MVP was initiated in the cluster in 2006, there were 16 health posts (case de santé) and one primary health care clinic (PHC) (poste de santé). Working with government health bodies at local and regional levels, four other PHC clinics were added by 2010, strategically located throughout the cluster, to strengthen the local health system infrastructure and allow geographic access. Van Lerberghe et al. argue in a recent Lancet series that expanding coverage of health facilities is the first step to strengthening health systems for maternal survival, which is followed by scaling up human resources [25].

PHC clinics were staffed by a minimum of one nurse (*Infirmière*) and one midwife (*Sage-femme*), and designed to provide a comprehensive package of services, including facility-based deliveries supported by a midwife. The smaller health posts were designed to offer more basic health services under the auspices of a nurse or lay CHW minimally-trained in obstetric care. An extensive referral network, intended to achieve a continuum of care, was set-up. This involved basic ambulances to link households and health posts to PHCs, and an equipped ambulance service to link PHCs to the nearest hospital, located approximately seven kilometers away from the MVP site, outside of the study area boundary. All households were covered by routine CHW program activities including pregnancy, birth and death tracking at the household level.

The main caregivers of women who died within childbearing age were approached and invited to participate in VASA. Once consent was obtained, a trained fieldworker conducted the VASA interview. There were five identified pregnancy-related deaths during 2011, with ages ranging from 15–39. Clinical staff members caring for women whose deaths occurred at the hospital were not formally interviewed as part of the VASA process. Some of these staff may have later participated in hospital quality review sessions and subsequent quality improvement efforts. CHWs, local MVP site team health staff, community members, hospital staff, government health staff, West Africa Regional MVP staff, and New Yorkbased MVP staff were all involved at various levels of analysis within the rapid cycle of change review process.

### Data collection

Within the MVP area, CHWs are each assigned approximately 120 households, for which they provide basic and preventative household-level healthcare. Initially using paper-based data collection and management tools, CHWs transitioned to mHealth platform Childcount + in 2010. ChildCount + was developed by MVP to empower communities to improve child and maternal survival [26]. The platform uses SMS text messages or smartphone applications to facilitate and coordinate the activities of CHWs. Childcount + modules can be used for screening and treating common conditions of diarrhoeal disease, febrile illness and acute malnutrition, as well as vital statistics data collection, and monitoring of CHW workload and performance. The database includes built-in reminders, allowing CHWs to track antenatal and postnatal care visits, growth monitoring and immunization coverage within their households [27]. CHW managers monitored Childcount + data across the site with support from the wider health team and regional MVP staff. Periodic retraining of CHWs was conducted to optimize accuracy of data collection. All households within the study site were registered with Childcount+, allowing reliable tracking of pregnancies, births and deaths.

Death notification was done by CHWs immediately after identifying a death. For all deaths of children under five and women of aged 12-49 yo, a fieldworker trained to handle the sensitive nature of death and mourning in a culturally appropriate manner visited the household approximately one to two weeks after a death to meet with a family member or primary caregiver. If the caregiver gave consent, the fieldworker conducted a detailed interview using the MVP standardized VASA tool. The MVP VASA tool is based on the WHO verbal autopsy questionnaire, referred to by Leitao et al. [13], but with some modifications that include an expanded section exploring social contributors to mortality such as access to transport. Information collected included demographic profile of the pregnant woman and interviewee, experiences of the woman during pregnancy such as health events and healthcare sought and used, and social circumstances around the death. During 2011, the site was transitioning from paper to mHealth collection of VASA so data were collected either by hand or using a mobile device. Regardless of collection method, data was subsequently uploaded to a central database. Once data had been uploaded, a pre-set algorithm was used to determine probable cause of death and contributing social factors. Clinical and project staff were able to access the completed questionnaire via the database at any time for clarification or further detailed analysis including age, place of death, gestation and parity.

### Data analysis

As part of the MVP quality improvement efforts, site teams are encouraged to review routinely collected health data, and to conduct monthly morbidity and mortality meetings in which de-identified VASA findings are discussed alongside other routine health indicators such as childhood growth monitoring. Input from community members is sought at these meetings. Trends of maternal deaths over time are collated on an annual and semi-annual basis, and outcomes across multiple sites reviewed by regional MVP staff. Through these routine processes, it was noted in mid-2011 there seemed to be an increase in maternal deaths in our study site. Health team staff revisited the cases and reviewed the circumstances around these deaths in more detail.

The dual system of paper and electronic versions of VASA and algorithm results were crosschecked and verified by clinicians for plausibility and accuracy. Clarification was sought from CHWs, clinic staff or household members if required. Each case of maternal death was summarized and tabulated to examine patterns. Once it was determined that preventable factors may have been involved in several maternal deaths, a comprehensive report was put together. Identifiers were removed to ensure anonymity of the presented data, given the sensitive nature of this case study. This report was then presented to target audiences on the basis of need and relevance, largely involving individuals or groups most likely to redress problems, such as the regional medical director, hospital director and hospital clinical manager. Other members of the multidisciplinary MVP intervention team were also provided with summary results for input. Feedback from these meetings was captured as part of the quality improvement cycle, and for future reference and utilization. The process was deliberately reiterative, with multiple cycles of review and analysis, and new data added as it became available.

### Ethics

Ethical approval for data collection using Childcount + and VASA tools was granted by the National Ethics Committee for Health Research in Senegal (Senegal Comite National d’Ethique pour la Recherche en Sante – NERS). Permission for the study was also obtained from the Ministry of Health (Direction de la Sante) in Senegal. Written informed consent was sought from all VASA interviewees prior to data collection. However, consent for publication of raw data was not obtained from caregivers of women tracked following maternal deaths, because data was collected as part of routine care. Furthermore, publication of the dataset presents minimal risk to confidentiality of study participants because the dataset has been rendered anonymous.

## Results

The results section of the study is presented in two parts: firstly, case analysis of all maternal deaths in the MVP cluster, and secondly, interventions followed in response to a rise in maternal mortality.

### Case analysis

**Figure 2:**
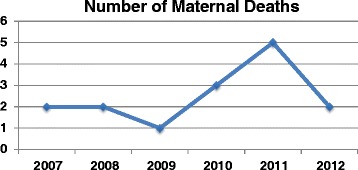
**Trends in the number of maternal deaths from 2007 to 2012.**

The number of maternal deaths in the MVP cluster located in Senegal was monitored since 2007. There were two or less recorded maternal deaths per year in 2007–2009, rising to three in 2010 and five in 2011 (See Figure 2). The deaths in 2011 led to an intensified approach to the analysis of factors leading to mortality, and a series of meetings were held to address the problems flagged. In 2012, only two maternal death cases occurred, as shown in Figure 2. Table 1 presents a summary of the cases of maternal deaths in 2011. The Pathway to Survival framework was applied to identify points of weakness in the continuum of care. The case analysis below contains further descriptions on each case in the form of case summaries.

**Table 1:** **Case by case analysis of maternal deaths occurring in 2011 (n = 5)**

	**CASE 1**	**CASE 2**	**CASE 3**	**CASE 4**	**CASE 5**
Age of Mother (Years)	39	35	32	15	20
Gestational Age (Months)	9	5	9	9	5
Place of Childbirth	Home	Hospital	Hospital	Hospital	Hospital
Place of Death	Hospital	Hospital	Hospital	Hospital	Hospital
Cause of Death	PPH	PPH	PPH	PPH	Eclampsia
Pathway to Survival^#^	Level 1	Level 4	Level 4	Level 4	Level 4
	Level 2				

^#^Weaknesses in the Continuum of care using the Pathway to Survival Framework: Level 1 – Recognizing Illness or need for care; Level 2 – Seeking Assistance; Level 3 – Referring appropriately; Level 4 – Receiving adequate treatment.

#### Case summaries

##### Case 1

A 39-year old female at full-term, with a history of an attempt to hide her pregnancy, delivered vaginally at home with the help of a traditional birth attendant early in 2011. She walked to the clinic after delivery, but was immediately evacuated in an ambulance due to vaginal bleeding. She died at the hospital. The most probable cause of death was post-partum haemorrhage.

##### Case 2

A woman of 35 years of age was evacuated from the MVP cluster for an obstetric emergency to the hospital at five months of gestation. She received an emergency Caesarian section (C/S), but died at the hospital along with her baby in mid-2011. She died most likely due to post-partum haemorrhage following extensive bleeding.

##### Case 3

A 32-year old was evacuated in mid-2011 from her local clinic to the hospital for emergency obstetric care, but died from excessive bleeding during a C/S at full term of pregnancy. Her baby also died during the course of the delivery.

##### Case 4

A 15-year old young woman with a history of convulsions died in the latter part of 2011 at nine months of pregnancy, following referral from the cluster to the hospital. She was also operated on for C/S, and died after an episode of post-partum haemorrhage.

##### Case 5

A woman of 20 years of age from the MVP cluster died during normal birth in late 2011 at the referral hospital, most probably as a result of Eclampsia. She was only five months pregnant, had previously received traditional medicines, and later referred to the hospital by the local clinic.

All five cases of maternal deaths occurred in the hospital. Four of the deaths occurred as a result of haemorrhage, three of which followed a surgically assisted C/S. Notably, two of the women succumbed at only five months of pregnancy. Furthermore, case two and three, both of which took place in the same month, were associated with foetal or neonatal deaths, as was case five. Case one was a home birth, but death occurred in the hospital, and case five was due to a cause other than excessive bleeding. Therefore, the initial steps in the pathway to survival, such as home deliveries, care in the clinic and emergency evacuation, were less of a problem compared to the quality of care at the hospital.

### Interventions

The MVP team in Senegal observed in mid-2011 that two deaths occurred in the hospital. A meeting was requested with the hospital authorities to highlight the deaths. A confidential mortality audit was instituted, and a government delegation visited the hospital to further investigate the deaths. A meeting was therefore held with the regional medical director, who cited the problems in the hospital to be logistical and incidental, but regrettable. Following the deaths later in the year, a meeting was held between the clinicians from MVP and the hospital. The increasing mortality trend from the community-level mortality reviews was presented to the hospital, rising from an estimated 67/100000 in 2009, 202/100000 in 2010 and 392/100000 in 2011, albeit numbers too small for a robust statistical analysis. The VASA cases were used as a demonstration to locate the problem, which was largely in the hospital.

The joint team adopted a systematic process to identify bottlenecks and opportunities for quality improvement in the hospital, following another death from post-partum haemorrhage of a 20-year old woman who arrived with a full-term pregnancy at the hospital in early 2012 for emergency obstetric care. Problems were identified along with proposed interventions. These problems were largely related to resource shortage to support surgical care, including inadequate surgical packs and related obstetric emergency drugs, human resources to support post-operative care, technically skilled staff for surgery, lack of a blood bank and blood donors when needed, and other patient monitoring equipment. Some of the quick-win solutions implemented were provision of surgical packs, equipment and drugs by MVP, reshuffling, training and supervision of the surgical team by the hospital, and a combined effort to track, avail and prepare blood donors for obstetric emergency care.

Soon after these interventions were agreed upon, another death took place in the hospital, after a C/S. A wider meeting was held between the hospital, including administrative heads, and the MVP team, including regional representatives of MVP. The agreement was to move forward rapidly on the implementation of achievable resolutions in the short-term, and a discussion about sustainable solutions broke out as it was clear that much more than quick wins was needed to preserve the lives of women delivering in the hospital, particularly in relation to surgical care.

The more sustainable solutions agreed upon included quarterly mortality reviews, the overhaul of the hospital infrastructure by an international donor in 2013, the scale up of MVP services beyond the intervention zone, and outreach services by the hospital staff to support clinic-based midwives. Notably, as Figure 2 shows, one more death occurred in 2012, a woman who died at home after a clinic-based nurse inappropriately sent her back home – a failure in the second level of the Pathway to Survival. The death served to escalate a sense of urgency to existing problems, as a reminder that much more is needed to reduce maternal deaths, and the need for further sustainable solutions. The overall death rate in 2012 showed trends of decline when compared to 2010 and 2011, although the reduction could not be attributed exclusively to the quick win interventions employed.

## Discussion

In order to reduce maternal mortality, we first need to understand who, where, when, how, and why deaths occur within a community [7]. Accurate mortality data capture at the community level is the initial step in this process, and health system innovators in sub-Saharan Africa are attempting to build community-level vital registration systems [28]. In our study site, mortality tracking methodology evolved over time. Strategies used to ensure accuracy of mortality data included CHW coverage and registration of all households in the cluster, routine tracking of pregnancies and births, VASA for deaths of all women of reproductive age and utilisation of mHealth tools. Investigations of maternal deaths using a verbal autopsy tool that included social and environmental contributors facilitated an in-depth exploration of contributors to mortality. The combination of community-wide accurate mortality tracking and in-depth exploration of deaths gives a representative indication of contributors to maternal mortality within the study community. Although the circumstances of this case study were specific to a Senegal experience, the findings demonstrate five key lessons for the reduction of maternal deaths in resource-poor settings that may be widely applicable.

Firstly, understanding the full continuum of maternal care incorporated in the Pathway to Survival is useful for identification of potential health care inefficiencies and subsequent targeting of interventions. Secondly, routine household visits by CHWs create a platform for community-level surveillance of maternal health through pregnancy, birth and death tracking. Embedding tools for systematic data collection into these forms of routine care is achievable, even with only a small number of data points. Thirdly, use of mobile devices and electronic databases can enhance the timeliness of data collection and analysis, allowing rapid-cycle change to occur in real-time, and with input from a greater number of collaborators. Fourthly, when a problem is identified, or a source thereof, cooperation and pooling of resources between various actors in the health system can serve to foster multifaceted interventions needed to timely mitigate against such problems as an acute rise in maternal deaths. Closing the feedback loop is essential and regularly scheduled feedback meetings can encourage timely turnaround action. Finally, a sudden appearance of a problem may not necessarily occur as a result of a cause that is amenable to a quick solution as shown in this case study, but a combination of short- and long-term interventions may be necessary to remedy such a situation.

Maternal deaths occur at home or at the hospital, but they may also occur at the clinic or en route to a referral facility, as previously demonstrated in the Pathways to Survival framework [14]. While many of these deaths may result from obstructed labour or puerperal sepsis, post-partum haemorrhage (PPH) is still a leading cause of maternal deaths despite advances in management, as was demonstrated in our study [29,30]. Normally, the higher one ascends in the health system hierarchy and continuum of care, the better the resource capability to manage such complications as PPH [31]. Therefore, lower levels of the health system, including households for patients delivering in their homes, are generally ill equipped to manage obstetric complications. Hence, a well-structured patient referral network is critical to successful management of obstetric emergencies, largely by avoiding delays [32]. However, a challenge arises when this system is not optimized, for example, a referral hospital that is poorly equipped to manage surgical emergency obstetric care [33], and thereby hamper the natural progression of care in the pathways of survival. Such circumstances may increase rates of mortality as well as patient dissatisfaction and distrust, which may encourage under-utilization of the health system, potentially leading to increased home deliveries. Furthermore, system failures like these may necessitate measures such as usage of drugs ideally reserved for higher levels of the health system, for example misoprostol for prevention of PPH, to be used in the lower levels of care, such as households [34].

Routine surveillance at a household level is emerging as a priority to improve health conditions, particularly in resource-limited settings [35]. Household surveillance, demonstrated here in the form of pregnancy, birth and death notification by CHWs using both paper and mobile devices, can potentially create a platform for real-time monitoring, trigger early response to obstetric emergencies, and facilitate early evacuation of complicated deliveries. However, such community-based programs require optimal support from well-functioning clinic and hospital services, as well as proper referral networks and facilities [36]. When these community-based programs are equipped with VASA, they can potentially promote community participation in mitigating maternal death events through increased awareness and mortality reviews, thereby confronting both obstetric and social factors contributing to the risk of death [14]. Furthermore, VASA tools can be used for community-level public health diagnosis, which can in turn be used to mobilize the necessary resources, and as was done in this case study, to rapidly advocate for prioritization of health problems in an evidence-informed manner.

## Conclusions

The problem identified in this case study, a rise in maternal deaths, necessitated a collaborative multi-stakeholder effort for redress, leveraging on the short-term quick wins to alleviate severity, but also long-term sustainable initiatives. Joint planning between governmental and non-governmental authorities and providers through a rapid cycle change model enabled successful implementation of quick wins, which appear to have limited the number of hospital-associated maternal deaths in the subsequent 12-month period. As to whether these combined efforts will be sustained to achieve long-term benefits or fall through the cracks following the initial success, still remains to be seen. Additional work is still needed to strengthen the continuum of care, maximize the benefits of community-based surveillance of deaths supported with VASA, and further reduce the risk and impact of maternal deaths. With the small numbers of death reported in this study, calculating maternal mortality rate reliably remains a challenge. However, the observed trends are suitable for the purposes of rapid cycle change model required for quality improvement. The routine nature of data collection, without rigorous verification, cannot exclude undetected cases of maternal deaths, particularly early pregnancy states. Within the constraints of small numbers in this study and a resource-poor environment, we can conclude that VASA is a suitable model for rapid identification and analysis of potential problems, as well as engaging with stakeholders. In this study, VASA results served to trigger action and sensitize both practitioners and decision-makers in an effort to achieve an early response to maternal deaths, and potentially contribute to reduction in the rate of maternal mortality**.**

## Abbreviations

CHW: Community health worker
MDG: Millennium development goal
mHealth: Mobile health
MMR: Maternal mortality ratio
MVP: Millennium villages project
PHC: Primary health care
C/S: Caesarian section
PPH: Post- Partum haemorrhage
RAMOS: Reproductive age mortality studies
VA: Verbal autopsy
VASA: Verbal and social autopsy
WHO: World Health Organisation

## References

[1] World Health Organisation (2014). Trends in Maternal Mortality: 1990 to 2013.

[2] Hogan MC, Foreman KJ, Naghavi M, Ahn SY, Wang M, Makela SM, Lopez AD, Lozano R, Murray CJL (2010). Maternal mortality for 181 countries, 1980–2008: a systematic analysis of progress towards Millennium Development Goal 5. Lancet.

[3] Lozano R, Wang H, Foreman KJ, Rajaratnam JK, Naghavi M, Marcus JR, Dwyer-Lindgren L, Lofgren KT, Phillips D, Atkinson C, Lopez AD, Murray CJL (2011). Progress towards Millennium Development Goals 4 and 5 on maternal and child mortality: an updated systematic analysis. Lancet.

[4] Helleringer S, Duthé G, Kanté AM, Andro A, Sokhna C, Trape J, Pison G (2013). Misclassification of pregnancy‐related deaths in adult mortality surveys: case study in Senegal. Trop Med Int Health.

[5] Garces RG, Sobel HL, Pabellon JA, Lopez JM, de Quiroz M, Nyunt-U S (2012). A comparison of vital registration and reproductive-age mortality survey in Bukidnon, Philippines, 2008. Int J Gynaecol Obstet.

[6] United Nations Population Fund (2011). The State of the World’s Midwifery 2011: Delivering, Health, Saving Lives.

[7] Ronsmans C, Graham WJ, Lancet Maternal Survival Series Steering Group (2006). Maternal mortality: who, when, where, and why. Lancet.

[8] Pirkle CM, Dumont A, Traoré M, Zunzunegui M (2013). Effect of a facility-based multifaceted intervention on the quality of obstetrical care: a cluster randomized controlled trial in Mali and Senegal. BMC Pregnancy Childbirth.

[9] Pattinson R, Kerber K, Waiswa P, Day LT, Mussell F, Asiruddin SK, Blencowe H, Lawn JE (2009). Perinatal mortality audit: counting, accountability, and overcoming challenges in scaling up in low-and middle-income countries. Int J Gynaecoy Obstet.

[10] Pirkle CM, Dumont A, Zunzunegui M (2011). Criterion-based clinical audit to assess quality of obstetrical care in low-and middle-income countries: a systematic review. Int J Qual Health Care.

[11] Homer C, Clements V, McDonnell N, Peek M, Sullivan E (2009). Maternal mortality: what can we learn from stories of postpartum haemorrhage?. Women Birth.

[12] Streatfield PK, Wasif A, Khan WA, Bhuiya A, Alam N, Sié A, Soura AB, Bonfoh B, Ngoran EK, Weldearegawi B, Jasseh M, Oduro A, Gyapong M, Kant S, Juvekar S, Wilopo S, Williams TN, Odhiambo FO, Beguy D, Ezeh A, Kyobutungi C, Crampin A, Delaunay V, Tollman SM, Herbst K, Chuc NTK, Sankoh OA, Tanner M, Byass P (2014). Pregnancy-related mortality in Africa and Asia: evidence from INDEPTH Health and Demographic Surveillance System sites. Glob Health Action.

[13] Leitao J, Chandramohan D, Byass P, Jakob R, Bundhamcharoen K, Choprapawon C (2013). Revising the WHO verbal autopsy instrument to facilitate routine cause-of-death monitoring. Glob Health Action.

[14] Kalter HD, Salgado R, Babille M, Koffi AK, Black RE (2011). Social autopsy for maternal and child deaths: a comprehensive literature review to examine the concept and the development of the method. Popul Health Metr.

[15] Thaddeus S, Maine D (1994). Too far to walk: maternal mortality in context. Soc Sci Med.

[16] Claeson M, Waldman RJ (2000). The evolution of child health programmes in developing countries: from targeting diseases to targeting people. Bull World Health Organ.

[17] Waldman RJ, Bartlett AV, Campbell CC, Steketee RW (1996). Overcoming Remaining Barriers: the Pathway to Child Survival.

[18] Pronyk PM, Muniz M, Nemser B, Somers MA, McClellan L, Palm CA, Huynh UK, Ben Amor Y, Begashaw B, McArthur JW, Niang A, Sachs SE, Singh P, Teklehaimanot A, Sachs JD (2012). The effect of an integrated multisector model for achieving the Millennium Development Goals and improving child survival in rural sub-Saharan Africa: a non-randomised controlled assessment. Lancet.

[19] Remans R, Pronyk PM, Fanzo JC, Chen J, Palm CA, Nemser B, Muniz M, Radunsky A, Abay AH, Coulibaly M, Mensah-Homiah J, Wagah M, An X, Mwaura C, Quintana E, Somers MA, Sanchez PA, Sachs SE, McArthur JW, Sachs JD (2011). Multisector intervention to accelerate reductions in child stunting: an observational study from 9 sub-Saharan African countries. Am J Clin Nutr.

[20] Geynisman J, Latimer A, Ofosu A, Anderson FW (2011). Improving maternal mortality reporting at the community level with a 4-question modified reproductive age mortality survey (RAMOS). Int J Gynaecol Obstet.

[21] Mechael P, Cruz-Cunha M, Tavares A, Simoes R (2010). Opportunities and Challenges of Integrating mHealth Applications into Rural Health Initiatives in Africa. Handbook of Research on Developments in E-Health and Telemedicine: Technological and Social Perspectives.

[22] Agarwal S, Labrique A (2014). Newborn health on the line: the potential mHealth applications. J Am Med Assoc.

[23] Graham WJ, Hussein J (2006). Universal reporting of maternal mortality: an achievable goal?. Int J Gynaecol Obstet.

[24] Cox S, Wilcock P, Young J (1999). Improving the repeat prescribing process in a busy general practice. a study using continuous quality improvement methodology. Qual Health Care.

[25] Van Lerberghe W, Matthews Z, Achadi E, Ancona C, Campbell J, Channon A, de Bernis L, De Brouwere V, Fauveau V, Fogstad H, Koblinsky M, Liljestrand J, Mechbal A, Murray SF, Rathavay T, Rehr H, Richard F, ten Hoope-Bender P, Turkmani S (2014). Country experience with strengthening of health systems and deployment of midwives in countries with high maternal mortality. Lancet.

[26] Berg M, Wariero J, Modi V (2009). Every Child Counts: The Use of SMS in Kenya to Support the Community-Based Management of Acute Malnutrition and Malaria in Children under Five.

[27] Singh P, Sachs JD (2013). 1 million community health workers in sub-Saharan Africa by 2015. Lancet.

[28] Prata N, Gerdts C, Gessessew A (2012). An innovative approach to measuring maternal mortality at the community level in low-resource settings using mid-level providers: a feasibility study in Tigray, Ethiopia. Reprod Health Matters.

[29] Rueda-Clausen CF, Campbell J, Baker PN (2011). Current challenges in pregnancy-related mortality. Obstet Gynaecol Reprod Med.

[30] Khan KS, Wojdyla D, Say L, Gülmezoglu AM, Van Look PF (2006). WHO analysis of causes of maternal death: a systematic review. Lancet.

[31] Dogba M, Pierre F, Dumont A, Zunzunegui M, Tourigny C, Berthe-Cisse S (2011). Mother and newborn survival according to point of entry and type of human resources in a maternal referral system in Kayes (Mali). Reprod Health.

[32] Ganyaglo GY, Hill WC (2012). A 6-year (2004–2009) review of maternal mortality at the Eastern Regional Hospital, Koforidua, Ghana. Semin Perinatol.

[33] De Brouwere V, Dieng T, Diadhiou M, Witter S, Denerville E (2009). Task shifting for emergency obstetric surgery in district hospitals in Senegal. Reprod Health Matters.

[34] Prata N, Graff M, Graves A, Potts M (2009). Avoidable maternal deaths: three ways to help now. Glob Public Health.

[35] McCaw-Binns A, Lindo JL, Lew-Bell KN, Ashley DE (2008). Maternal mortality surveillance in Jamaica. Int J Gynaecol Obstet.

[36] Bahl R, Qazi S, Darmstadt GL, Martines J (2010). Why is continuum of care from home to health facilities essential to improve perinatal survival?. Semin Perinatol.

